# Development and Validation of a Short Measure of Emotional, Physical, and Behavioral Markers of Eustress and Distress (MEDS)

**DOI:** 10.3390/healthcare10020339

**Published:** 2022-02-10

**Authors:** Helen Pluut, Petru L. Curșeu, Oana C. Fodor

**Affiliations:** 1 Department of Business Studies, Leiden University, 2311 ES Leiden, The Netherlands; h.pluut@law.leidenuniv.nl; 2 Department of Psychology, Babeş-Bolyai University, 400015 Cluj-Napoca, Romania; oanafodor@psychology.ro; 3 Department of Organization, Open Universiteit, 6419 AT Heerlen, The Netherlands

**Keywords:** distress, eustress, performance, well-being, measurement, MEDS

## Abstract

We report the results of three validation studies for a short measure of emotional, physical, and behavioral markers of eustress and distress as they occur when individuals encounter stressful events in academic and organizational settings. Given the importance of the distinction between “positive” and “negative” stress as well as the recent resurgence of research exploring the differences between challenge and hindrance stress and between eustress and distress, it is important to put forward a short, validated scale that evaluates these constructs. Our short measure—the MEDS—therefore has important theoretical as well as practical implications. By showing that the eustress and distress subscales have adequate internal consistency and good construct and criterion validity, we open new avenues for research that extends our knowledge and understanding of the antecedents and consequences of eustress and distress. We also discuss appropriate uses of the scale in educational and organizational settings.

## 1. Introduction

Occupational stress among employees and academic stress among students have been of increasing concern in today’s society due to their deleterious effects on well-being and performance [1,2,3,4,5]. Researchers have therefore focused extensively on psychological stress, and there is considerable consensus among scholars that stress should be understood in a context, that is, as a relationship between a person and the environment. The cognitive-transactional model of Lazarus [6] captures this relational view, as it defines stress as a process in which environmental demands tax or exceed the person’s coping resources. Through primary appraisal, the person evaluates to what extent the environmental demands (i.e., stressors) pose harm, threats, or challenges [7]. While threat involves an anticipation of harm or losses, challenge appraisals result from feeling positive about a demanding encounter because it has potential for gain and growth. Thus, stress is an individual outcome generated at the interface between individual and situational factors, which is reflected through the person’s appraisal of the environmental demands. Because a stressful encounter can be appraised as either threatening (negative) or challenging (positive) and because “stress is a post-appraisal state” [8] p. 4, it follows that two different types of stress can be distinguished: distress (dysfunctional or “bad stress”) and eustress (functional or “good stress”).

The concept of eustress finds its roots in positive psychology and organizational behavior. Selye [9,10] was the first to use the term when he differentiated between distress and eustress. Selye conceptualized stress as a non-specific response to a stressor with either negative or positive effects, and he termed these effects distress and eustress, respectively. Although his work has led to some confusion among stress scholars, Selye’s conceptualization of distress and eustress as effects or outcomes has been highly influential [11]. The work of Lazarus [6] and Lazarus and Folkman [7] on stress appraisal, however, marked a change in researchers’ conceptions of (eu)stress. The stress response was no longer seen as non-specific and unidimensional but rather as a complex process involving appraisal. A person differentiates stressors as either threatening or challenging, and the occurrence of distress and eustress is determined by this appraisal of stressors. As such, eustress should be understood “as a positive response to a cognitively appraised stressor” [11] p. 279, which is reflected in emotional, physical, and behavioral domains [6].

Research has argued that there is consistency in the way particular stressors are appraised by the individuals who are exposed to them [12], in that we can distinguish between challenge and hindrance stressors [13]. Challenge stressors refer to work-related situations that are generally appraised as promoting growth and learning, whereas those aspects of the job that are viewed as obstacles to personal development and accomplishment are categorized as hindrance stressors. An important assumption underlying the challenge–hindrance stressor framework of occupational stress is that the pattern of challenge versus hindrance appraisals in reaction to certain stressors is assumed to be the same for all employees [14]. More specifically, previous research has assumed that some stressors (e.g., workload, responsibility, time urgency) are uniformly experienced as challenges, while other stressors (e.g., role ambiguity, resource inadequacies, organizational politics) are viewed as hindrances [12,15]. However, the transactional theory of stress [6,7] suggests that an a priori classification of stressors as challenges or hindrances is reductionist and simplistic. Indeed, studies have shown that stressors can be cognitively appraised as both challenges (“eustressing”) and hindrances (“distressing”) simultaneously [3,14,16]. Thus, although we concur with the broad categories in the two-dimensional stressor framework, personal reactions to stressful encounters are likely to differ. Therefore, in order to disentangle eustress and distress (i.e., challenge-based and hindrance-based stress), one needs to evaluate the markers for the two complex psychological states in the emotional, physical, and behavioral domains. 

Although there are numerous scales for the assessment of distress (amongst others, the widely used Perceived Stress Scale by Cohen et al. [17]), eustress has not received enough attention in terms of operationalization and measurement. In a recent paper, Merino et al. [18] used positive and negative affect experienced in relation to COVID-19 quarantine as indicators of eustress and distress, respectively. The COVID-19 pandemic is indeed a global stressor [19,20,21,22] that can trigger either eustress or distress in the population [20,23]. We have put forward a more comprehensive multidimensional approach of measuring eustress and distress in academic and organizational settings by taking into consideration emotional, physical, and behavioral indicators that discriminate between the two complex psychological states. In other words, we have distinguished between eustress and distress as different ways people can react to a particular stressor, and we have developed the markers of eustress and distress to capture the emotional, physical, and behavioral markers of eustress and distress. This approach is in line with the conceptualization of stress as a response that follows from the interaction between personal and situational variables. As previous operationalizations of eustress [24,25] rather reflected outcomes of the stress response, it seems that the dominance of the theoretical perspective in the role of appraisal in the stress process [6,7] has not been accompanied by the development of theory-based psychometric instruments for measuring markers of eustress. This paper aims to address this issue, as we have developed and tested a brief measure—the MEDS, for assessing the emotional, physical, and behavioral markers of eustress and distress in academic and organizational settings.

## 2. Defining Eustress and Distress

In line with Lazarus [6] we see stress as a multidimensional response to a stressor that is manifested in psychological, physiological, and behavioral terms. This response can be described as either eustress or distress or a combination of both [26]. We define eustress as a constructive type of stress that reflects a state of positive emotional arousal associated with activation and engagement. Distress is a destructive type of stress and reflects a state of negative emotional arousal associated with dissatisfaction and disengagement. The eustressful or distressful nature of a particular stressor is dependent on a number of factors [3,26]. As can be seen in Table 1, we have identified four dimensions that differentiate eustress and distress. First of all, the amount of demand imposed on the individual by the stressor distinguishes between eustress and distress. Eustress is associated with moderate levels of demands, whereas distress is associated with low and high levels of demands. This is in line with the Yerkes–Dodson Law, which argues that people perform best at moderate levels of arousal (compared to low and high levels), and Quick et al. [27] used the term eustress to describe this optimal quantity of stress. The second dimension on which eustress and distress differ is the degree of control one has over the stressful situation. Karasek’s [28] Demands–Control Model shows that a lack of control is likely to result in distress. Control as a situational belief is part of the appraisal process [7] and is therefore likely to influence whether a person experiences eustress (average to high control) or distress (little to no control). Finally, eustress and distress differ in the extent to which a stressor is appraised as a threat or a challenge [29]. Threat and challenge appraisals form two separate dimensions and can occur simultaneously as responses to the same stressor [7]. Eustressing situations are appraised as challenging but not threatening, whereas distressing situations are appraised as threatening but not challenging.

## 3. Pilot Study: MEDS Development—Content Validity

Lazarus [6] claimed that the stress response is psychological (affective), physiological, and behavioral in nature. For the development of our self-report measure, we started from this notion of stress as a three-dimensional concept, which has been empirically overlooked so far. In terms of psychological manifestations, eustress is reflected in positive emotional reactions, and distress is reflected in negative emotional reactions. Physiologically, eustress is manifested in feeling energized, vigorous, and activated, whereas distress is associated with feeling physically drained. Finally, eustressed and distressed people differ in their behaviors and behavioral intentions in the sense that eustressed people are eager to perform, whereas distressed people show impaired performance (e.g., unfocused and error prone). We developed a pool of items measuring eustress and distress on each of the three dimensions. Example items for eustress were “I feel excited” (emotional), “I sense I am physically in shape” (physical), and “I am ready to act!” (behavioral). For distress, items included “I feel nervous” (emotional), “I sense I am exhausted” (physical), and “I am not able to concentrate on my work/study” (behavioral).

We pretested this list of 18 items in a group of 10 independent experts to evaluate the content adequacy of the items. The experts were presented the MEDS items in random order. We provided them with definitions of eustress and distress and then asked them to distribute the items to the appropriate subscales (i.e., eustress and distress). The results of the sorting task are shown in Table 2, and it can be seen that the experts’ categorization of the items was correct in 98.9% (178 of 180) of the cases. We could therefore conclude that the items were reflective of the constructs they intended to measure.

## 4. Study 1: Experiment with Scenarios Testing the Discriminant Validity of the MEDS

In order to test the construct validity of the MEDS, we decided to perform an experiment using a scenario-based approach. The experiment required respondents to fill out the scale after reading two scenarios that were supposed to independently trigger eustress and distress in academic settings. In order to find a discriminating pair of scenarios, we started by developing four sets of paired scenarios related to (a) being late for an exam, (b) intragroup conflict, (c) approaching a deadline for an assignment, and (d) an evaluation talk with a study advisor. These situations describe stressful academic events [3] that our respondents could easily relate to. For each of the stressors, one scenario was expected to induce (mostly) eustress and the other distress. The eustress scenarios were intended to capture challenging situations with a moderate degree of demand and a high sense of control, whereas the distress scenarios should have described threatening situations with high demands and a low degree of control. We pre-tested those scenarios to select the most discriminative pair for the experiment. We asked nine Masters and PhD students enrolled at a Dutch university to evaluate the four paired scenarios in terms of the level of distress and eustress they would experience and in terms of each of the dimensions mentioned above, that is, amount of demand, degree of control, threat, and challenge (each of the six evaluative dimensions was shortly described in order to make it comprehensible). Afterwards, we had a short debriefing session, in which we explained the purpose of the scenarios, and the respondents provided us with feedback and suggestions for improving the scenarios in such a way that these would be highly discriminative between eustress and distress. 

We analyzed the paired scenarios by means of Generalized Linear Model Repeated Measures in SPSS version 24 (IBM, Armonk, NY, USA). Based on preliminary inspection of the results, in the first pair of scenarios, we observed a rather substantial contamination between distress and eustress, in the sense that the “planned” eustress scenario was rated as being highly distressful as well. This situation was dropped from further explorations. For the other three paired scenarios, the contamination of eustress and distress in the eustress scenario was limited. Nevertheless, the patterns of perceived degree of control, demand, and challenge did not match the expected patterns for two eustress scenarios, which were also dropped from further analysis. 

**Figure 1:**
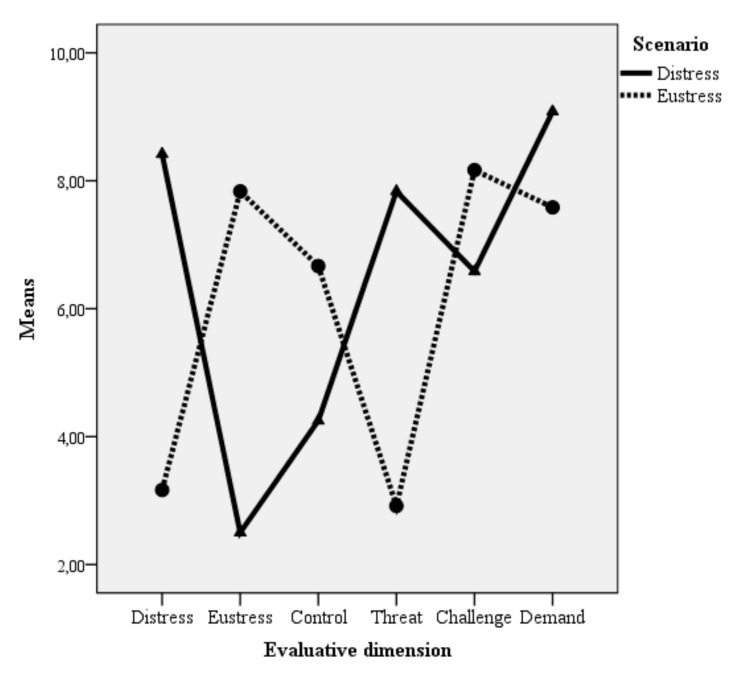
Results of the cross-validation for the eustress and distress scenarios. Note: all paired comparisons per dimension between the two scenarios were statistically significant, with the exception of challenge, where the difference (although in the expected direction) was not statistically significant.

The distinction in the remaining paired scenario reflected two common types of intragroup conflict as discussed in the literature, namely task conflict (i.e., disagreements about the task) and relationship conflict (i.e., interpersonal frictions) [30]. Intragroup conflict is a stressful event, yet task and relationship conflict are two qualitatively different stressors [31]. The eustress scenario of intragroup conflict represented a situation in which the focal person experienced task conflicts, which were uneasy at times but held the potential to improve the group performance (i.e., a reasonable amount of control). In contrast, the distress scenario placed respondents in a situation of relationship conflict, which was interpersonal in nature and interfered with effective group work. The results pointed to the situations describing conflicts related to group work as discriminative with respect to the eustress/distress dimension. We used a mixed model ANOVA with the eustress/distress evaluation as a within-subject factor and the type of scenario as a between-subject factor, and the results showed a significant interaction effect between the within- and the between-subject factors, F(1, 16) = 9.01, *p* = 0.008, η² = 0.36 (the observed power was π = 0.80), illustrative of the discriminative nature of the two scenarios. With respect to the other evaluative dimensions, the task conflict scenario was perceived to have a higher degree of control, F(1, 16) = 6.49, *p* = 0.02, η² = 0.29 (the observed power was π = 0.67), and to be less threatening, F(1, 16) = 12.67, *p* = 0.003, η² = 0.44 (the observed power was π = 0.91), than the relationship conflict scenario. The results for perceived challenge and amount of demand, however, did not clearly differentiate the two scenarios. Based on these results and on the basis of the respondents’ feedback (e.g., the distress scenario was not too stressful and some still felt in control), we made small adjustments to the texts to increase the discriminative nature of the scenarios (i.e., reducing the amount of challenge and control and increasing the demands present in the distress scenario). The adjusted pair of scenarios that was used for the validation study is presented in the Supplementary Materials. Because we planned to carry out the actual scale validation study in a different cultural context, we cross-validated the adjusted pair of scenarios by asking 12 students enrolled at a large Romanian university to evaluate the two adjusted scenarios on the same six dimensions as in the preliminary phase. The results of the cross-validation fully fitted our expectations, namely that the distress scenario scored high on distress, threat, and demand, while the eustress scenario scored high on eustress, challenge, and control. The results are presented in Figure 1.

The adjusted pair of scenarios was used to test the discriminant validity of the eustress and distress scales in a sample of 74 students (64 women, average age of 22.43 years) enrolled at a large Romanian university. Participants were first presented with one scenario (either task or relationship conflict) and were asked to read the text and identify as much as possible with the situation described and then evaluate their reaction to the situation using the eustress–distress scale. After this, they were presented with the other scenario and were again asked to fill out the scale. The response options accompanying the scale ranged from 1 = strongly disagree to 5 = strongly agree. In this within-subjects design, the order in which the scenarios were presented to the respondents was fully randomized (i.e., 37 respondents received the task conflict scenario first, whereas 37 respondents received the relationship conflict scenario first).

The scales showed high reliability, with a Cronbach’s alpha for the eustress scale of 0.80 in the task conflict condition and 0.83 in the relationship conflict condition, while for the distress scale, it was 0.90 in the task conflict condition and 0.88 in the relationship conflict condition. In order to test the validity of the scales for eustress and distress, we analyzed whether the manipulation intended by the scenarios was reflected in the scores from the eustress–distress scale. More specifically, we expected that the task conflict scenario would trigger eustress, whereas the relationship conflict scenario was supposed to trigger distress. We used a repeated measures ANOVA and compared the eustress and distress evaluations across the two scenarios. Moreover, we compared the eustress and distress scores within each scenario. When comparing eustress scores across scenarios, we found that the eustress scale differentiated well between the two scenarios, as the eustress score was significantly higher for the task conflict scenario (M = 5.31, SD = 0.69) than for the relationship conflict scenario (M = 4.56, SD = 0.88), F(1, 70) = 41.79 (*p* < 0.001), η² = 0.37. Furthermore, the distress scale discriminated well between the two scenarios, as the distress score for the task conflict scenario (M = 3.14, SD = 1.06) was significantly lower than for the relationship conflict scenario (M = 5.13, SD = 0.96), F(1, 71) = 167.30, *p* < 0.001), η² = 0.69. Moreover, our results showed good discriminative power of the two scales for the within-scenarios comparison. Contrasting the scores of eustress and distress for each of the scenarios, we found that in the task conflict scenario (in the pretest associated with eustress), respondents scored significantly higher on eustress than on distress, F(1, 73) = 144.05, *p* < 0.001), η² = 0.66. For the relationship conflict scenario (in the pretest associated with distress), the eustress score was significantly lower than the distress score, F(1, 70) = 10.14, *p* = 0.002, η² = 0.12. Overall, our scales discriminated appropriately between, as well as within, the two scenarios, and we can, therefore, conclude that the results provide support for the discriminative power and validity of our eustress and distress scales.

## 5. Study 2: Field Study Using a Student Sample Assessing Construct and Criterion Validity of the MEDS

In this second study, we (1) tested the discriminant and convergent validity of the MEDS through the eustress–distress intercorrelation and the correlation with measures of related constructs in an academic context, and we (2) tested the predictive validity of the MEDS, with academic performance on an exam as the criterion variable. A sample of 280 students (47.3% female) that were enrolled in a bachelor course at a Dutch university participated in the study. We assessed their levels of eustress and distress towards the end of an evaluation period that occurred halfway through the semester. We used the eustress–distress scale in conjunction with two other commonly measured constructs in the well-being domain, namely engagement (i.e., a positive fulfilling state) and perceived stress (i.e., experiencing life as uncontrollable and overloaded). The measures that we used to evaluate those constructs have been validated and widely used in research. We measured study engagement with the 9-item Utrecht Work Engagement Scale (UWES) [32], which we adapted to a higher education context (e.g., “When I study, I feel bursting with energy”). Stress was measured with 10 items from the Perceived Stress Scale by Cohen and colleagues [17]. All answers were recorded on a five-point Likert scale ranging from 1 = strongly disagree to 5 = strongly agree. Cronbach’s alpha for the study engagement scale was 0.75, and we found an alpha of 0.84 for perceived stress. At the end of the semester, students performed a knowledge test consisting of 60 multiple choice questions. 

We checked the reliability of the MEDS and found a comparable Cronbach’s alpha for the distress scale (0.84) but a lower value for the eustress scale (0.65) compared to the values reported in Study 1. We expected that eustress, as a state of positive arousal that is associated with activation, would relate positively to engagement and negatively to perceived stress. Distress refers to a state of negative arousal associated with dissatisfaction and was therefore expected to correlate positively with perceived stress and negatively with engagement. Correlation analyses (see Table 3) showed the expected convergent pattern between eustress and distress on the one hand and engagement and perceived stress on the other hand. Eustress related positively to engagement (*p* < 0.001) and negatively to perceived stress (*p* = 0.013), while distress showed a negative but not significant correlation with engagement (*p* = 0.557) and a positive correlation with perceived stress (*p* < 0.001). Importantly, the positive correlations were modest, demonstrating that the scales measured overlapping but conceptually distinct concepts. These findings provide support for the convergent validity of our scales. 

We then tested the predictive validity of the MEDS by relating it to a criterion variable assessed at a later point in time, namely an objective measure of academic performance (Table 4). We expected that students that scored high on eustress were in a state of activation and motivated and would therefore score higher on the knowledge test compared to students who scored low on eustress. We also expected that the negative state of distress would negatively predict academic performance. To test these claims, we conducted a stepwise regression analysis, in which we entered engagement and perceived stress in Step 1 and added eustress and distress in Step 2. The results indicated, first of all, that perceived stress negatively predicted performance on the exam (*p* = 0.023). Adding eustress and distress to the model, we found that eustress had a significant positive effect (*p* = 0.009), and distress had a strong negative effect (*p* < 0.001) on academic performance. Thus, as expected, eustress and distress predicted academic performance in opposing ways. Interestingly, the effect of perceived stress that was found in Step 1 became not significant when we added eustress and distress in Step 2 (*p* = 0.658). It can be concluded that our markers of eustress and distress were stronger or more proximal predictors of academic performance than general feelings of stress and engagement. It must be noted that we found only a weak relation between eustress and distress (see Table 3, *p* = 0.073), which showed that these are constructs that share only a limited amount of variance. This quasi-independence is an attractive feature of the MEDS, as eustress and distress appear to be orthogonal constructs that both explain a significant amount of variance in the criterion.

## 6. Study 3: Field Study Using an Organizational Sample Assessing Construct and Criterion Related Validity of the MEDS

To further assess the psychometric properties of the MEDS using a nonstudent sample, we collected data in an organizational context. The MEDS evaluates emotional, behavioral, and physical markers of eustress and distress, and given these multiple evaluative dimensions, we expected that the eustress and distress scores should explain additional variance in a relevant criterion that was left unexplained by constructs that captured a single evaluative dimension. Specifically, we expected that our eustress subscale could explain additional variance in work–family enrichment beyond positive affect, while the distress subscale could explain additional variance in work–family conflict beyond negative affect. In this final study, we distributed our eustress–distress scale among 62 employees (situated in the Netherlands and the USA) of a global company that supplies machines and systems for the food processing industry. The survey also included the Positive Affect Negative Affect Schedule [33] as a measure of related constructs to test the convergent validity as well as scales for work–family conflict [34] and work–family enrichment [35] as criteria to test the concurrent validity of our eustress–distress scale. The values of the Cronbach’s alpha for all scales are shown in Table 5 along with the correlational matrix. We found high reliabilities for our eustress (0.84) and distress (0.84) scales in this study.

We expected positive correlations between eustress and positive affect (as both scales capture positive states at work) and between distress and negative affect (as both scales capture negative states at work). The correlations, as presented in Table 5, show that eustress had a significant positive association with positive affect (*p* < 0.001) as well as a significant negative correlation with negative affect (*p* = 0.017), while distress correlated negatively with positive affect (but not significantly, *p* = 0.164) and positively with negative affect (*p* < 0.001). Thus, the correlational findings supported the convergent validity of our eustress and distress scales, as they correlated modestly in the expected direction with the respective affective items in our scale. 

Previous research has found a link between trait affectivity and the work–family interface in such a way that negative affect (and not positive affect) is associated with work–family conflict, while positive affect (and not negative affect) is associated with work–family enrichment [36,37]. We extended this logic to state (rather than trait) affect and expected that eustress and distress related to work–family enrichment and conflict, respectively. Correlational analysis showed that, in line with the previously mentioned studies, positive work affect was positively associated with work–family enrichment (*p* = 0.011), and negative work affect was positively correlated with work–family conflict (*p* < 0.001). Interestingly, work–family enrichment was more strongly linked to eustress (*p* = 0.001) than to positive affect, and work–family conflict was more strongly linked to distress (*p* < 0.001) than to negative affect. Stepwise regression analyses for work–family conflict and enrichment revealed a similar pattern (see Table 6). 

More specifically, distress was strongly associated with work–family conflict (*p* < 0.001), and eustress explained a significant amount of variance in work–family enrichment (*p* = 0.020). Controlling for eustress and distress in Step 2, positive and negative affect did not significantly relate to the work–family outcomes, although we found significant associations between negative affect and work–family conflict (*p* < 0.001) and between positive affect and work–family enrichment (*p* = 0.011) in Step 1. These results provide support for the concurrent validity of the MEDS and show that due to their multidimensional nature, eustress and distress evaluations with the MEDS have explanatory power above and beyond positive and negative affect with respect to work–family outcomes.

## 7. Item Validation and Dimensionality of the MEDS—Factorial Validity

Another step in the validation of the MEDS was to test whether the items captured the hypothesized underlying dimensions, i.e., eustress and distress. Respondents’ answers for the 18 items were factor analyzed in the datasets from Study 1 and Study 2. We used a principal component analysis (PCA) and extracted a fixed number of two factors. The two factors were then rotated using the direct Oblimin rotation method. The factor loadings for the 18 items in the resulting pattern matrices are presented in Table 7. 

The majority of items had strong primary loadings on the hypothesized factor, with acceptable low secondary loadings. The factor loadings pointed to the emotional markers of eustress as somewhat problematic. Nevertheless, the 18 items in total seemed to adequately capture the underlying dimensions of eustress and distress and could therefore be said to be good indicators of their corresponding constructs. The factors representing eustress and distress were only weakly correlated (r = −0.02 in Study 2 and r = −0.21 in Study 3), again supporting the construct independence. 

Building on the validation of the items, we performed a confirmatory factor analysis using structural equation modeling to confirm the dimensionality of the MEDS. Our set of 18 items was intended to differentiate between eustress and distress, and we therefore, first of all, compared the two-factor model with a single factor model that would represent a general stress component. The model fit of the single factor model was inferior to that of the two-factor model, indicating that eustress and distress are distinct constructs. We then examined a six-factor model, with three items reflecting each of the six dimensions. In the large student sample from Study 2, the principal factor analysis extracted six components based on eigenvalues above 1 that together accounted for 70.2% of the variance. This is in line with Lazarus’ [6] conceptualization and our operationalization of eustress and distress as three-dimensional constructs. More specifically, both types of stress have emotional, physical, and behavioral markers. Analysis of this six-factor model confirmed the hypothesized factor structure, as the fit indices pointed to the model with six factors as the best fitting model. Although this demonstrates the multidimensionality of the stress constructs, the subdimensions represented markers of the same construct, and we therefore suggest that researchers consider using the total nine-item scores as indicators of eustress and distress. An overview of the nested model comparisons as well as fit statistics for each of the models is presented in Table 8.

## 8. Discussion

We have argued that the distinction between distress and eustress is conceptually meaningful but has received little attention in terms of operationalization and measurement both in academic and organizational settings. Development of a multidimensional stress measure is therefore a much-needed step. We have presented three validation studies for the MEDS, which consists of 18 items that capture emotional, physical, and behavioral markers of eustress and distress. We based the development of our items on Lazarus’ [6] theoretical notion of stress as a three-dimensional construct, and we validated the developed scale in multiple studies using diverse samples. The following steps were taken to validate the MEDS. Content validity was assessed by asking experts to sort the 18 items to one of the subscales. A factor analysis confirmed the hypothesized two-factor structure of the eustress–distress scale. The construct validity and discriminative power of the subscales were tested in a scenario-based experiment in Study 1. Through the significant but modest correlations between our eustress and distress measures on the one hand and related constructs on the other hand, we showed the convergent validity of the scale in Study 2 and Study 3. These studies also provided support for the predictive and concurrent validity of the MEDS through the correlational patterns of eustress and distress with their respective external criteria. Finally, the values for Cronbach’s alpha revealed good reliability of the MEDS in all three studies. Overall, we can conclude that the MEDS developed in this research represents a short, reliable, and valid measure that captures emotional, physical, and behavioral markers of two types of stress responses. 

Our scale could provide further theoretical refinement of eustress and distress and allows for the development of integrative models of the two constructs. One plausible research direction is to test the convergent validity with other measures of eustress and distress in the literature. In doing so, one could reveal whether it is a focus on the stressor, the appraisal, or the stress outcome that yields more discriminative results for eustress and distress. Such a convergent investigation could also show whether the integrative focus on multiple dimensions (i.e., emotional, physical, and behavioral markers of the stress response) yields higher predictive validity than the focus on a single evaluative dimension, as was the case with the scales developed by Branson et al. [38] and Rodriguez et al. [16]. Our third study offered relevant insights into the added value of using multiple markers of eustress and distress. Here, we showed that eustress and distress scores predicted variance in two relevant and often-studied constructs, work–family conflict and enrichment, beyond the affective evaluative dimension of well-being. Valid measures of eustress and distress can add to our understanding, especially regarding the positive outlook on stress. In line with the dominant view in the literature [9,10,11], we conceptualized eustress as a beneficial appraisal tendency that fosters effective adaptation to stressors. Eustress could, however, also have detrimental effects in the longer term. After all, eustress is still stress from which one has to recover. As the beneficial or detrimental nature of a reaction to a stressor can only be evaluated after the consequences are known, future research could explore, in a longitudinal manner, how distress and eustress influence well-being, adaptation, and resilience over time. Moreover, also from a theoretical perspective, valid evaluation scales for eustress and distress enable valuable avenues of research into the antecedents of the two constructs. Future research could explore the role of personal (e.g., general self-efficacy [39]) and interpersonal resources (e.g., social support), as well as of other contextual factors (e.g., the salience of the stressor) [40], that can differentially trigger a distress or eustress appraisal of a particular stressor. 

The MEDS can be immensely practical for managers to evaluate the level of eustress and distress in an organization, as the two appraisal tendencies are likely to impact employee performance and well-being. A specific area of application for such a scale is situations of organizational development and change. Planned organizational change is an impactful event [41] that can trigger appraisals as distress or eustress and, as such, can influence the extent to which employees embrace change and engage proactively with the new developments or, in contrast, resist change and engage in counterproductive work behaviors. As organizational leaders and managers are key change actors in organizational settings [42], they can proactively assess employees’ evaluative tendencies towards planned organizational change and gain a clear understanding of resistance to change in order to manage such complex change processes more effectively. 

The MEDS could also be used by educational researchers and prove useful for educators and professionals, especially those working in higher education settings, to evaluate markers of the two types of stress that have the potential of explaining academic performance and student well-being. In the second study, we showed that the two stress types predict academic performance in a university student sample. Moreover, in line with the results of studies 2 and 3 that eustress and distress are negatively correlated, one would expect that participants would tend to appraise a stressor distinctively as generating either eustress or distress. Such an insight is relevant for educational settings, where study coaches and advisors could use the scale to swiftly evaluate the way in which students appraise particular stressors and design specific supportive interventions to alleviate the negative consequences of distress. In this context, it may also be interesting to focus on students that score high on both dimensions (i.e., eustress and distress). It is highly relevant for this group of students to understand the appraisal mechanisms that generate high scores for both distress and eustress in order to try to reinforce the evaluative processes that support eustress appraisal. Future research could explore the cognitive evaluative processes that explain the appraisal of a particular stressor as generating eustress or distress. 

The COVID-19 pandemic offers a particularly relevant context in which antecedents of eustress and distress can be reliably explored, also in cross-cultural studies, especially due to the global nature of the pandemic. A specific area of application of the short measure of eustress and distress is to better understand humans’ adaptation to the COVID-19 pandemic [21,22,40]. On the one hand, it is clear that the constant focus of the media and public discourse on the threats associated with the pandemic generate ruminative tendencies that predict self-handicapping tendencies and exhaustion [43], ultimately reducing wellbeing [40,44,45]. On the other hand, the use of humor in interpersonal communication in relation to the pandemic reduces anxiety and negative mood in the context of the COVID-19 pandemic [20]. In other words, it generates an appraisal context in which the pandemic is less likely to be perceived as threatening and therefore stressful. Certainly, such an appraisal tendency may also have negative consequences when it tends to reduce the likelihood of engaging in protective behaviors [19]. In the context of the COVID-19 pandemic, we would expect that constant rumination about the dangers of the pandemic leads to distress and protective behaviors, while the use of humor in communication about COVID-19 leads to eustress. It would also be fruitful to understand how students adapted to disrupted educational routines and to what extent the eustress and distress appraisals are important mechanisms that explain academic adaptation and satisfaction. All in all, we hope that the MEDS will spur research on the antecedents and consequences of eustress and distress in the context of the COVID-19 pandemic.

### Limitations

While this study makes important contributions, several limitations should also be noted. First, we draw attention to the rather small sample used in Study 3, which limits the generalizability of our results and also may have affected the results of the factor analysis, especially the fit indices reported for our models. We carried out the validation studies in two cultural contexts (The Netherlands and Romania), and our results seemed to be robust in a cross-cultural context. However, future research should further explore the psychometric qualities of the scales in other organizational settings and cultural contexts. We call for more cross-cultural investigations on the two stress appraisal tendencies in order to add to our theoretical understanding of the two constructs. Second, we did not start our item selection with a large pool of items from which to select the final scale items based on factorial or item response analyses. However, we used a theory-driven scale development approach, and we selected the physical, emotional, and behavioral markers of eustress and distress with the highest face validity. Third, the scenario-based experiment used to validate the newly developed MEDS in Study 1 relied on newly developed scenarios that were designed to trigger eustress and distress appraisals. As both the scenarios and the MEDS were new, this could have affected the validation process of the scales. However, we relied on an expert-based evaluation of the scenarios and pilot tested them on the relevant dimensions discriminating between eustress and distress. 

## 9. Conclusions

Our study provides initial empirical evidence for the validity and reliability of a short scale to evaluate the emotional, physical, and behavioral markers of eustress and distress (MEDS). We built on a multidimensional perspective on stress to evaluate the emotional, behavioral, and physical markers of eustress and distress, and we argue that the MEDS presented here can be used in clinical, organizational, and educational settings. Our approach was aligned with the transactional theory of stress [6,7] by focusing on the multidimensional nature of stressor appraisal rather than the nature of the stressor itself, therefore allowing the differential measurement of eustress and distress as distinguishable dimensions of stressor appraisal. To conclude, the MEDS is a theory-derived and empirically validated measure of eustress and distress markers.

## Figures and Tables

**Table 1:** Dimensions of Eustress and Distress.

Dimension	Eustress	Distress
Amount of demand	Moderate	Low or high
Degree of control	Average to high control	Little or no control
Threat appraisal	Low threat	High threat
Challenge appraisal	High challenge	Low challenge

**Table 2:** Overview of Items and Expert Distributions.

Item	Eustress	Distress
I feel excited.	100%	0%
I feel determined.	100%	0%
I feel alert.	90%	10%
I feel nervous.	0%	100%
I feel tense.	10%	90%
I feel irritated.	0%	100%
I sense I am energetic.	100%	0%
I sense I am physically in shape.	100%	0%
I sense I am full of life.	100%	0%
I sense I am fatigued.	0%	100%
I sense I am exhausted.	0%	100%
I sense I am drained.	0%	100%
I am ready to act!	100%	0%
I am willing to expand efforts on my study/work.	100%	0%
I am prepared to engage in my study/work.	100%	0%
I am not able to concentrate on my study/work.	0%	100%
I cannot handle my study/work problems.	0%	100%
I make lots of errors in my daily study/work.	0%	100%

**Table 3:** Descriptive statistics and correlations for variables included in Study 2.

	M	SD	1	2	3	4
1. Eustress	3.36	0.44	1			
2. Distress	2.51	0.66	−0.13 ^†^	1		
3. Engagement	2.98	0.48	0.40 **	−0.04	1	
4. Perceived stress	2.80	0.61	−0.16 *	0.56 **	−0.13 *	1
5. Exam performance	62.6	10.52	0.19 **	−0.29 **	−0.02	−0.14 *

Note: ^†^ *p* < 0.10. * *p* < 0.05. ** *p* < 0.01.

**Table 4:** Stepwise Regression Results for Academic Performance (Study 2).

Step	Independent Variables	Model 1	Model 2
1	Engagement	0.00 (0.03)	−0.05 (0.03)
	PSC	−0.15 * (0.02)	0.03 (0.02)
2	Eustress		0.18 ** (0.03)
	Distress		−0.29 *** (0.02)
	F F change	2.695 2.695	7.139 *** 11.347 ***

Note. PSC = Perceived Stress Scale; standardized regression coefficients are shown with standard errors between parentheses; * *p* < 0.05. ** *p* < 0.01. *** *p* < 0.001.

**Table 5:** Descriptive Statistics and Correlational Matrix of the Study 3 Variables.

	M	SD	1	2	3	4	5	6
1. Eustress	3.94	0.43	(0.84)					
2. Distress	1.94	0.59	−0.30 **	(0.84)				
3. Positive affect	3.82	0.48	0.49 **	−0.18	(0.82)			
4. Negative affect	1.62	0.50	−0.30 *	0.68 **	−0.22 ^†^	(0.79)		
5. Work-family conflict	2.48	0.72	−0.29 *	0.66 **	0.02	0.43 **	(0.86)	
6. Work-family enrichment	3.44	0.63	0.41 **	−0.39 **	0.32 **	−0.28 **	−0.49 **	(0.92)

Note. Internal consistency values are shown in between brackets on the diagonal. ^†^ *p* < 0.10. * *p* < 0.05. ** *p* < 0.01.

**Table 6:** Stepwise Regression Results for Work–Family Conflict and Enrichment (Study 3).

		Work-Family Conflict		Work-Family Enrichment	
Step	Independent Variables	Model 1	Model 2	Model 1	Model 2
1	Negative Affect	0.43 *** (0.17)	−0.03 (0.19)		
	Positive Affect			0.32 * (0.16)	0.16 (0.18)
2	Distress		0.68 *** (0.16)		
	Eustress				0.33 * (0.20)
	F	13.585 ***	22.476 ***	6.836 *	6.564 **
	F change	13.585 ***	25.684 ***	6.836 *	5.743 *

Note. Standardized regression coefficients are shown with standard errors between parentheses. * *p* < 0.05. ** *p* < 0.01. *** *p* < 0.001.

**Table 7:** Pattern Matrices from Exploratory Factor Analyses.

	Study 2		Study 3	
Item Wording	Eustress	Distress	Eustress	Distress
I feel excited.	**0.21**	−0.56	**0.64**	−0.18
I feel determined.	**0.37**	−0.29	**0.19**	−0.25
I feel alert.	**0.51**	0.30	**0.58**	−0.13
I feel nervous.	0.13	**0.62**	0.30	**0.80**
I feel tense.	0.19	**0.66**	0.31	**0.75**
I feel irritated.	0.06	**0.76**	−0.20	**0.51**
I sense I am energetic.	**0.52**	−0.39	**0.75**	−0.14
I sense I am physically in shape.	**0.60**	−0.01	**0.75**	0.03
I sense I am full of life.	**0.63**	−0.09	**0.84**	−0.03
I sense I am fatigued.	0.10	**0.68**	0.30	**0.58**
I sense I am exhausted.	0.00	**0.76**	0.31	**0.67**
I sense I am drained.	0.03	**0.69**	−0.20	**0.74**
I am ready to act!	**0.61**	−0.07	**0.83**	0.08
I am willing to expand efforts on my study/work.	**0.52**	0.28	**0.65**	0.38
I am prepared to engage in my study/work.	**0.51**	0.11	**0.44**	−0.18
I am not able to concentrate on my study/work.	−0.08	**0.52**	−0.01	**0.56**
I cannot handle my study/work problems.	−0.05	**0.64**	−0.08	**0.68**
I make lots of errors in my daily study/work.	−0.07	**0.59**	0.01	**0.63**

Note. The pattern matrices follow from a Principal Component Analysis with the direct Oblimin rotation method. The hypothesized factor structure is marked in bold.

**Table 8:** Nested Model Comparisons in the Confirmatory Factor Analysis.

Model	Chi-Square (*df*)	TLI	CFI	RMSEA	AIC	Chi-Square Difference Test
Study 2						
M1: 1 factor	979.9 (135)	0.32	0.46	0.150	1087.9	-
M2: 2 factors	863.5 (134)	0.41	0.54	0.140	973.5	M2-M1 = 116.4 ***
M3: 6 factors	363.5 (120)	0.78	0.85	0.085	501.5	M3-M2 = 499.9 ***
Study 3						
M1: 1 factor	462.8 (135)	0.22	0.38	0.196	570.8	-
M2: 2 factors	332.7 (134)	0.52	0.63	0.153	442.7	M2-M1 = 128.1 ***
M3: 6 factors	215.2 (120)	0.74	0.82	0.112	353.2	M3-M2 = 89.5 ***

*** *p* < 0.001.

## Data Availability

The datasets generated during and analyzed during the current study are available from the corresponding author on reasonable request.

## Supplementary Materials

The following are available online at https://www.mdpi.com/article/10.3390/healthcare10020339/s1, Scenarios used in Study 1.
