# Supplementary material for: Development and Validation of a Short Measure of Emotional, Physical, and Behavioral Markers of Eustress and Distress (MEDS)
Source: Healthcare (Basel). 2022 Feb 10;10(2):339. doi: 10.3390/healthcare10020339 (PMC8872528; doi:10.3390/healthcare10020339)
Supplement: Supplementary file 1 [file healthcare-10-00339-s001.zip › healthcare-1574390-supplementary.pdf]

Supplementary Materials

The Content of the Validation Scenarios used in Study 1

Page: 1

Eustress (task conflict scenario) - Imagine you are working together with five other students on a group project for one of the courses you have to pass. It is really important for you (personally) to deliver a good group project at the end of the semester. Throughout the project, there have been a lot of disagreements and discussions in the group. Although you do not always like the fact that your ideas are challenged, you realize that the group project might improve from debates in the group. You actually feel that the group has been able to come up with creative ideas and correct mistakes in some meetings that involved heavy discussions. You realize that disagreements can be in the benefit of the group, if you deal with it appropriately.

Distress (relationship conflict scenario) - Imagine you are working together with five other students on a group project for one of the courses you have to pass. It is really important for you (personally) to deliver a good group project at the end of the semester. Throughout the project, there have been a lot of quarrels and personal attacks in the group. As the deadline approaches, the tensions and fights with your colleagues become extreme. The situation is getting out of hand! You are very frustrated because you cannot resolve these conflicts. The assignment is far from ready, and your colleagues obstruct you from focusing on what needs to be done. You realize the group project will certainly fail.
